# A Lusus Naturae

**Published:** 1863-03

**Authors:** E. Collins


### Correspondence.

### A LUSUS NATURÆ.

BY E. COLLINS.

I have in my possession a first superior molar tooth which I extracted for a lady a year or eighteen months ago which presents the following peculiarities :

*First.* On the external or buccal surface are the remains of a supernumerary crown, which was originally one-fourth the size of the crown proper.

*Second.* It has but one root which is curved and marked by a shallow groove posteriorly and anteriorly.

*Third.* About a line from the edge of the enamel on each side and situated in the center and terminating points of the grooves, and exactly opposite to each other are two beautiful globes of enamel of pearly whiteness, which are about the size of a millet seed, and which stand out in bold relievo resembling somewhat the eyes of the crawfish.

The several peculiarities possessed by this tooth give to it a striking resemblance in form to the head of a bird I have seen, but the name of which I do not now remember. The two former peculiarities I do not mention as uncommon, but, occurring in connection with the latter, certainly entitles the tooth to a place among the wonders of the world.

Query. How did this division and subdivision of the enamel take place during the formative procees ? Don't all answer at once.

